# Development of machine learning prognostic models for overall survival of prostate cancer patients with lymph node-positive

**DOI:** 10.1038/s41598-023-45804-x

**Published:** 2023-10-27

**Authors:** Zi-He Peng, Juan-Hua Tian, Bo-Hong Chen, Hai-Bin Zhou, Hang Bi, Min-Xin He, Ming-Rui Li, Xin-Yu Zheng, Ya-Wen Wang, Tie Chong, Zhao-Lun Li

**Affiliations:** 1 Department of Urology, The Second Affiliated Hospital of Xi’an Jiaotong University, Xi’an, Shaanxi China; 2 Department of Urology, The First Affiliated Hospital of Xi’an Jiaotong University, Xi’an, Shaanxi China; 3 Health Science Center, Xi’an Jiaotong University, Xi’an, Shaanxi China

**Keywords:** Prostate cancer, Prostate cancer

## Abstract

Prostate cancer (PCa) patients with lymph node involvement (LNI) constitute a single-risk group with varied prognoses. Existing studies on this group have focused solely on those who underwent prostatectomy (RP), using statistical models to predict prognosis. This study aimed to develop an easily accessible individual survival prediction tool based on multiple machine learning (ML) algorithms to predict survival probability for PCa patients with LNI. A total of 3280 PCa patients with LNI were identified from the Surveillance, Epidemiology, and End Results (SEER) database, covering the years 2000–2019. The primary endpoint was overall survival (OS). Gradient Boosting Survival Analysis (GBSA), Random Survival Forest (RSF), and Extra Survival Trees (EST) were used to develop prognosis models, which were compared to Cox regression. Discrimination was evaluated using the time-dependent areas under the receiver operating characteristic curve (time-dependent AUC) and the concordance index (c-index). Calibration was assessed using the time-dependent Brier score (time-dependent BS) and the integrated Brier score (IBS). Moreover, the beeswarm summary plot in SHAP (SHapley Additive exPlanations) was used to display the contribution of variables to the results. The 3280 patients were randomly split into a training cohort (n = 2624) and a validation cohort (n = 656). Nine variables including age at diagnosis, race, marital status, clinical T stage, prostate-specific antigen (PSA) level at diagnosis, Gleason Score (GS), number of positive lymph nodes, radical prostatectomy (RP), and radiotherapy (RT) were used to develop models. The mean time-dependent AUC for GBSA, RSF, and EST was 0.782 (95% confidence interval [CI] 0.779–0.783), 0.779 (95% CI 0.776–0.780), and 0.781 (95% CI 0.778–0.782), respectively, which were higher than the Cox regression model of 0.770 (95% CI 0.769–0.773). Additionally, all models demonstrated almost similar calibration, with low IBS. A web-based prediction tool was developed using the best-performing GBSA, which is accessible at https://pengzihexjtu-pca-n1.streamlit.app/. ML algorithms showed better performance compared with Cox regression and we developed a web-based tool, which may help to guide patient treatment and follow-up.

## Introduction

Prostate cancer (PCa) is the second most common disease and the fifth major cause of cancer mortality among men in 2020, with an expected 1.4 million new cases and 375,000 deaths globally^1^. According to the American Cancer Society (ACS), in 2022, the number of new cases of PCa in the United States is expected to reach 268,490, accounting for 27% of all male malignancies, making it the most prevalent cancer, while the number of new deaths is expected to reach 34,500, accounting for 21% of all male malignancies, second only to lung and bronchus cancer^2^. The incidence and mortality rates of PCa in Asia manifest notably lower figures in comparison to their European and American counterparts. However, recent years have borne witness to a discernible ascendant trajectory, characterized by a swifter rate of ascent than observed within the developed nations of Europe and the United States^3, 4^. In the year 2020, the incidence of PCa in China is projected to reach 15.6 cases per 100,000 individuals, yielding a distressing tally of over 115,000 new diagnoses and an unfortunate toll of 51,000 lives lost to this affliction^1^. Lymph node involvement (LNI) is considered a single-risk group according to the National Comprehensive Cancer Network (NCCN) guidelines and European Association of Urology (EAU) guidelines^5, 6^. Traditional imaging suggests that about 5% to 10% of newly diagnosed PCa patients are suspected to have pelvic lymph node invasion without distant metastasis^6^. The incidence of pathological LNI (pN1) after radical prostatectomy (RP) varied from 0 to 37%, depending on the risk category and the regions excised during pelvic lymph node dissection (PLND)^7^. The prognosis of a PCa patient significantly deteriorates if LNI is detected, increasing the risk of tumor recurrence and mortality^8, 9^.

Given the variable prognosis of PCa patients with LNI, several research teams have endeavored to develop prognostic models for this cohort. Abdollah et al.^10^ developed a nomogram to predict cancer-specific mortality (CSM)-free survival in 1107 patients with LNI treated with RP and PLND. Another study subsequently conducted an external validation of Abdollah’s nomogram, which exhibited reduced predictive accuracy compared to internal validation (0.658 vs 0.833, respectively), with an area under the curve (AUC) of 0.667 (95% confidence interval [CI]: 0.601–0.730)^11^. Similarly, Hutten et al.^12^ developed prognostic nomograms for 336 patients with LNI after RP. The concordance index (c-index) for metastasis-free survival (MFS) and overall survival (OS) of the prognostic models were 0.85 and 0.71, respectively. Nonetheless, all of these models had a major drawback in that they only predicted the survival of patients after RP, and the initial treatment modalities for clinical LNI (cN1) patients included both surgical and non-surgical treatments, with limited evidence supporting the benefit of surgery for patients with LNI. Moreover, the inadequacy of the sample size and the uniformity of the prediction algorithms constrained the performance of these models.

The Cox regression has traditionally been used to develop prognostic models. However, this method assumes linearity, thereby impeding its capacity to depict the intricate, multidimensional, and nonlinear interplays among various prognostic factors inherent in biological systems. Therefore, its prognosis forecasting ability is limited. Conversely, machine learning (ML) algorithms exhibit numerous advantages over Cox regression, given that they employ nonlinear functions and account for all possible variable interactions to enhance predictive performance^13^.

Based on these premises, our study endeavored to develop prognostic models that predict OS in PCa patients with LNI (cN1 or pN1) through the utilization of three ML algorithms, alongside Cox regression, relying on a vast cohort. We present the following article in accordance with the TRIPOD reporting checklist (Supplementary Information)^14^.

## Methods

### Patient selection

**Figure 1:**
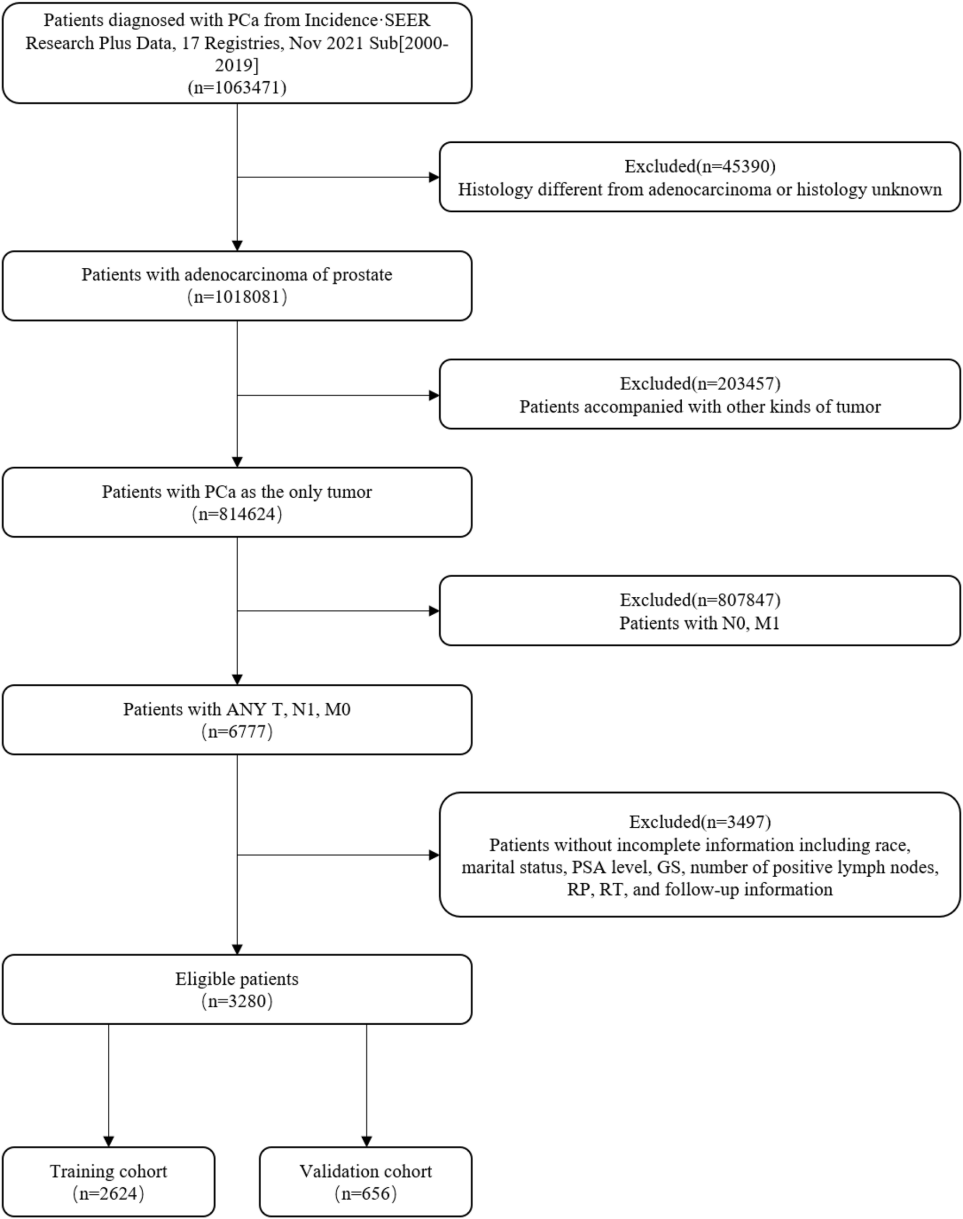
Flowchart outlining the inclusion and exclusion criteria for patients in the Surveillance, Epidemiology, and End Results (SEER) database from 2000 to 2019. *PCa* prostate cancer, *PSA* prostate-specific antigen, *GS* Gleason Score, *RP* radical prostatectomy, *RT* radiotherapy.

The present study utilized data obtained from the Surveillance, Epidemiology, and End Results (SEER) database, a publicly accessible dataset containing information from 18 population-based cancer registries. Using the SEER*Stat software (version 8.4.0), patients diagnosed with PCa (ICD-O-3 code: C61.9) between 2000 and 2019 were selected following the inclusion and exclusion criteria listed in Fig. 1. A total of 3524 non-metastatic patients with LNI satisfying the stated criteria were included in the study and subsequently randomly divided into a training and a validation cohort in an 8:2 ratio. The training cohort was utilized for the development of the model, while the validation cohort was used for the evaluation and validation of the model.

### Variable selection and endpoint

Demographic and clinical data for patients with PCa were extracted from the SEER database including age at diagnosis, race, marital status, clinical T stage, prostate-specific antigen (PSA) level at diagnosis, Gleason Score (GS), number of positive lymph nodes, RP, radiotherapy (RT) and follow-up information. Lymph node information for PCa patients (cN1 or pN1) was obtained from the Collaborative Stage Data Collection System Coding in the seer database. Based on previous literature, we grouped the variables into distinct categories^10, 12, 15^. Age was categorized as follows: ≤ 60 years, 61–69 years, and ≥ 70 years old. The clinical T stage was classified into T1-T3a, T3b, and T4. The PSA level, measured as a continuous variable ranging from 0.1 to 98.0 ng/mL, was recorded, with values of 98 ng/mL or greater noted as 98 ng/mL. The GS was categorized into ≤ 3 + 4, 4 + 3, 8, and ≥ 9. The number of positive lymph nodes was recorded as the exact number of regional lymph nodes examined by the pathologist and was subsequently categorized into 1, 2, and ≥ 3. RT included both initial and adjuvant treatment. “Survival months” and “Vital status recode” as outcome variables were extracted. The forward and backward stepwise selection was used to screen variables with prognostic values. The primary endpoint of interest was OS, which was calculated from the date of diagnosis to the date of death.

### Model development

Three ML algorithms including Gradient Boosting Survival Analysis (GBSA), Random Survival Forest (RSF), and Extra Survival Trees (EST) were used to develop prognostic models and compared Cox regression^16^. The model was iteratively tested and adjusted to determine the parameters of the best model. Model parameter settings were detailed in Supplementary Table 1.

### Model performance evaluation

The model's discrimination was evaluated using the time-dependent areas under the receiver operating characteristic curve (time-dependent AUC) and the c-index. Additionally, calibration was assessed using the time-dependent Brier score (time-dependent BS). Time points were selected within the 5th and 95th percentiles from the survival time distribution of the training and validation cohort. The integrated Brier score (IBS), which represents a cumulative BS over time, was also used to evaluate model performance. To estimate the reliability of the performance assessment, a 95% CI was calculated for each performance evaluation by bootstrapping a sample from the validation cohort 500 times.

### Model interpretation

To interpret ML models, we utilized the SHAP (SHapley Additive exPlanations, version 0.41.0) package in Python^17^. Specifically, we used the beeswarm summary plot in SHAP to display the contribution of variables to the results. SHAP is a game-theoretic methodology developed to explain the results generated by ML models. This approach can help identify which features are most important for the model's predictions and how they affect the model's output.

### Statistical analysis

To compare potential differences between the training, validation, and primary cohort, non-normally distributed continuous variables were evaluated using the Kruskal–Wallis test and reported as the median (interquartile range, IQR). Categorical variables were evaluated using the χ2 test and reported as frequencies (%). In the statistical analysis and model development, R (version 4.1.2, The R Foundation) and Python (version 3.9.12, Python Software Foundation) were utilized. All ML algorithms were developed based on scikit-survival (version 0.17.2). A p value of less than 0.05 was considered statistically significant.

### Ethics approval and consent to participate

The data from SEER is publicly available and de-identified, so no informed patient consent was required to release the SEER database. The ethics committee of the Second Affiliated Hospital of Xi’an Jiaotong University waived the need for ethical approval and informed consent.

## Results

### Patient characteristics

This study enrolled a total of 3280 eligible patients, with 2624 patients assigned to the training cohort and 656 patients assigned to the validation cohort. In the training cohort, 544 (20.7%) patients experienced mortality, while 2080 (79.3%) patients survived. The validation cohort had 134 (20.4%) deaths and 522 (79.6%) survivals. For further particulars concerning the patients, kindly refer to Table 1. Notably, there were no statistically significant differences observed in the variables between the training cohort, the validation cohort, and the primary cohort.

**Table 1:** Baseline characteristics of patients in the training cohort and the validation cohort.

Variables	Primary cohort	Training cohort	Validation cohort	p value
	(n = 3280)	(n = 2624)	(n = 656)	
Age, yr(%)				
≤ 60	1780 (54.3)	1415 (53.9)	365 (55.6)	0.957
61–69	792 (24.1)	637 (24.3)	155 (23.6)	
≥ 70	708 (21.6)	572 (21.8)	136 (20.7)	
Race, n(%)				
White	2642 (80.5)	2099 (80.0)	543 (82.8)	0.628
Black	442 (13.5)	364 (13.9)	78 (11.9)	
Other^a^	196 (6.0)	161 (6.1)	35 (5.3)	
Marital status, n(%)				
Married	2377 (72.5)	1906 (72.6)	471 (71.8)	0.912
Unmarried^b^	903 (27.5)	718 (27.4)	185 (28.2)	
Clinical T stage, n(%)				
T1–T3a	2847 (86.8)	2270 (86.5)	577 (88.0)	0.912
T3b	298 (9.1)	243 (9.3)	55 (8.4)	
T4	135 (4.1)	111 (4.2)	24 (3.7)	
PSA level, ng/ml				
Median [IQR]	12.1[7.0, 26.9]	12.0 [7.0, 26.2]	12.5 [7.1, 29.7]	0.695
GS, n(%)				
≤ 3 + 4	632 (19.3)	514 (19.6)	118 (18.0)	0.384
4 + 3	619 (18.9)	512 (19.5)	107 (16.3)	
8	823 (25.1)	640 (24.4)	183 (27.9)	
≥ 9	1206 (36.8)	958 (36.5)	248 (37.8)	
Number of positive lymph nodes, n(%)				
1	1490 (45.4)	1190 (45.4)	300 (45.7)	0.996
2	452 (13.8)	366 (13.9)	86 (13.1)	
≥ 3	530 (16.2)	427 (16.3)	103 (15.7)	
No lymph nodes were examined	808 (24.6)	641 (24.4)	167 (25.5)	
RP, n(%)				
Yes	2308 (70.4)	1848 (70.4)	460 (70.1)	0.988
No	972 (29.6)	776 (29.6)	196 (29.9)	
RT, n(%)				
Yes	1217 (37.1)	985 (37.5)	232 (35.4)	0.588
No/Unknown	2063 (62.9)	1639 (62.5)	424 (64.6)	
Follow-up time, yr				
Median [IQR]	68.0[53.0, 89.0]	68.0 [53.0, 89.0]	68.0 [54.0, 88.0]	1.000
Survival status, n(%)				
Alive	2602 (79.3)	2080 (79.3)	522 (79.6)	0.985
Dead	678 (20.7)	544 (20.7)	134 (20.4)	

*PSA* prostate-specific antigen, *IQR* interquartile range, *GS* Gleason Score, *RP* radical prostatectomy, *RT* radiotherapy.

^a^Other: American Indian/Alaska Native, Asian/Pacific Islander.

^b^Unmarried: Divorced, Separated, Single (never married), Widowed, unmarried.

### Multivariate Cox regression analysis

Age, race, marital status, clinical T stage, PSA level, GS, number of positive lymph nodes, RP, and RT were included in Cox regression model for multivariate analysis. The results of the multivariate analysis were shown in Table 2. To screen the variables with prognostic values, the forward and backward stepwise selection was employed. The results revealed that all the variables, except the PSA level, were selected. Nonetheless, in line with the relevant medical knowledge, we incorporated the PSA level into the development of the prognostic model.

**Table 2:** Multivariate Cox regression analysis in the training cohort.

Variables	Multivariate analyses	p value
	HR (95%CI)	
Age		
≤ 60	Reference	
61–69	1.236 (0.992–1.541)	0.059
≥ 70	1.707 (1.388–2.100)	< 0.001
Race		
White	Reference	
Black	0.928 (0.717–1.203)	0.574
Other	0.647 (0.437–0.959)	0.030
Marital status		
Married	Reference	
Unmarried	1.640 (1.365–1.969)	< 0.001
Clinical T stage		
T1–T3a	Reference	
T3b	1.230 (0.946–1.599)	0.122
T4	1.740 (1.285–2.358)	< 0.001
PSA level (ng/ml)	1.001 (0.998–1.004)	0.501
GS		
≤ 3 + 4	Reference	
4 + 3	1.358 (0.936–1.971)	0.108
8	1.866 (1.339–2.600)	< 0.001
≥ 9	3.087 (2.273–4.192)	< 0.001
Number of positive lymph nodes		
1	Reference	
2	1.284 (0.944–1.746)	0.111
≥ 3	1.631 (1.247–2.133)	< 0.001
No nodes were examined	–	–
RP		
Yes	Reference	
No	1.517 (1.037–2.219)	0.032
RT		
Yes	Reference	
No/unknown	1.538 (1.276–1.854)	< 0.001

*HR* hazard ratio, *CI* confidence interval.

### ML prognostic model development and performance evaluation

**Figure 2:**
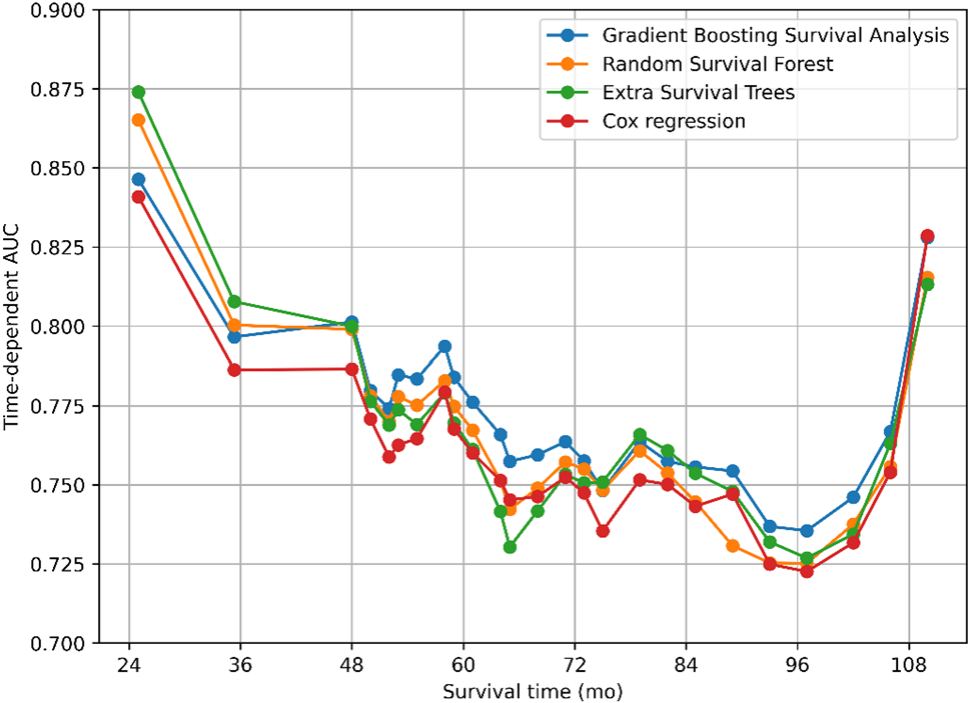
Time-dependent AUC for predicting overall survival (OS) of patients with various models including Gradient Boosting Survival Analysis (GBSA), Random Survival Forest (RSF), Extra Survival Trees (EST), and Cox regression.

**Figure 3:**
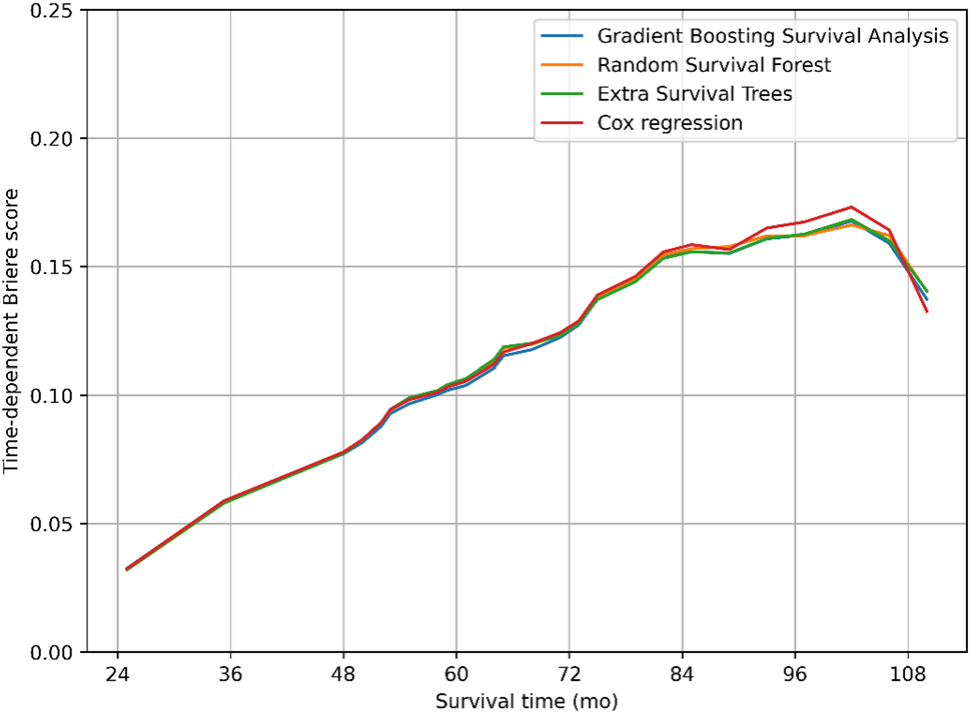
The prediction error curves for models including GBSA, RSF, EST, and Cox regression in predicting OS based on the time-dependent Brier score.

All variables were incorporated into prognostic models utilizing GBSA, RSF, EST, and Cox regression, respectively, to anticipate the OS of PCa patients with LNI. The time-dependent AUC for each model was presented in Fig. 2. GBSA, RSF, and EST exhibited a higher mean time-dependent AUC of 0.782 (95% CI: 0.779–0.783), 0.779 (95% CI: 0.776–0.780), and 0.781 (95% CI: 0.778–0.782), respectively, in comparison to Cox regression model with 0.770 (95% CI: 0.769–0.773). Correspondingly, the c-index of ML models surpassed that of Cox regression model with values of 0.745 (95% CI: 0.742–0.746), 0.743 (95% CI: 0.740–0.744), 0.745 (95% CI: 0.742–0.746), and 0.734 (95% CI: 0.732–0.736), respectively. Additionally, the prediction error curves founded on the time-dependent BS of the four models were exhibited in Fig. 3, with the four curves closely resembling each other. The integrated Brier score (IBS) for GBSA, RSF, EST, and Cox regression was calculated to be 0.114 (95% CI: 0.113–0.114), 0.114 (95% CI: 0.114–0.115), 0.114 (95% CI: 0.114–0.115), and 0.115 (95% CI: 0.115–0.116), respectively. No significant variance was observed in IBS between ML models and Cox regression model, and all models exhibited good calibration. The performance assessment of the models was succinctly summarized in Table 3.

**Table 3:** Model performance summary.

Models	Mean time-dependent AUC (95% CI)	C-index (95% CI)	IBS (95% CI)
GBSA	0.782 (0.779–0.783)	0.745 (0.742–0.746)	0.114 (0.113–0.114)
RSF	0.779 (0.776–0.780)	0.743 (0.740–0.744)	0.114 (0.114–0.115)
EST	0.781 (0.778–0.782)	0.745 (0.742–0.746)	0.114 (0.114–0.115)
Cox regression	0.770 (0.769–0.773)	0.734 (0.732–0.736)	0.115 (0.115–0.116)

*AUC* areas under the curve, *C-index* concordance index, *IBS* integrated Brier score.

### Interpretation of models

**Figure 4:**
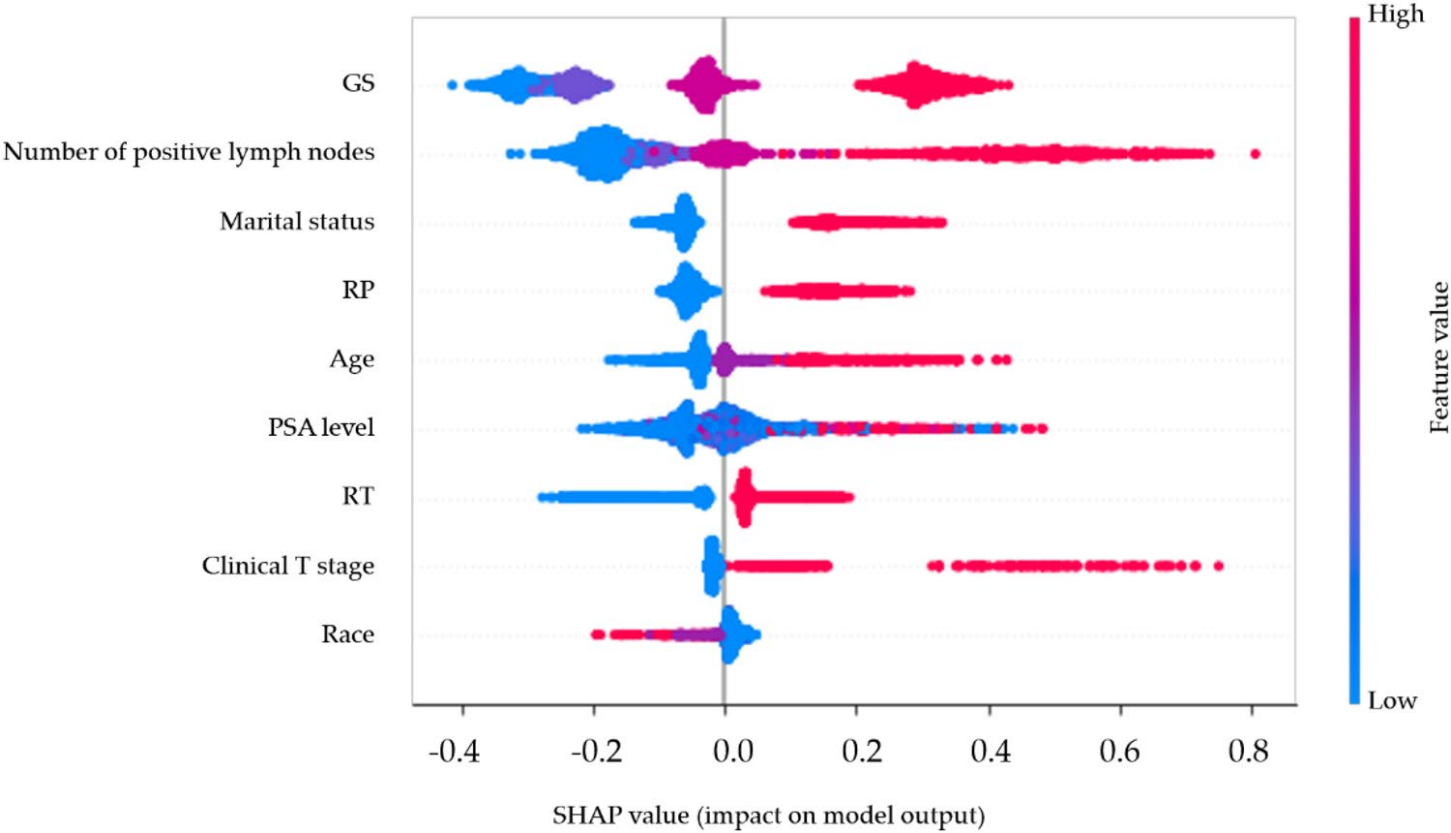
The beeswarm plot of GBSA model. It is an intricately designed graphical representation that offers a highly informative and information-dense summary of how the top features in a given dataset influence the model's output. Each point on the plot specifically refers to a distinct feature of a particular patient. The relative importance of each variable is depicted by its y position on the plot. Additionally, the SHAP value, which signifies the variable's contribution to the outcome, determines the x position of each dot, while dots accumulate along each feature row to demonstrate density. Color is used to showcase the original value of a feature. For example, the variable GS is the most significant risk factor, with an increased grade corresponding to a higher likelihood of poor prognosis.

ML models were visually interpreted. Within the beeswarm summary plot, model variables were arranged in descending order of importance. The GBSA model, which performed the best, revealed that the GS held the highest level of consequence, with the number of positive lymph nodes, marital status, RP, and age following suit, among other factors (Fig. 4). For beeswarm summary plots of other ML models, refer to Supplementary Fig. 1.

### Web predictor

Upon consideration of all performance evaluation metrics, GBSA model demonstrated the best performance. Consequently, an online predictor for forecasting OS in PCa patients with LNI was created based on GBSA algorithm. The survival curve and survival probability can be conveniently predicted by inputting the relevant variables on the web page (Supplementary Fig. 2; https://pengzihexjtu-pca-n1.streamlit.app/).

## Discussion

In this study, we conducted a comprehensive analysis of a large cohort of 3280 patients with LNI from the SEER database. Our ML models evinced superior discrimination in OS for patients compared with Cox regression model. Through the beeswarm summary plot for GBSA model, the GS was identified as the most significant risk variable, followed by the number of positive lymph nodes and marital status. Furthermore, the web-based individual prognostic tool based on the best-performing GBSA model showed potential in clinical practice. To our knowledge, this is the first ML prognostic model study for PCa patients with LNI.

There is a growing debate and increased interest surrounding the management of LNI PCa. With the continuous improvement of imaging technology, more and more PCa cases are being identified as LNI. Lymph node metastases were previously considered incurable and were exclusively treated with androgen deprivation treatment (ADT). However, emerging research suggested that those with LNI were likely to benefit even more from RP or RT. One systematic review including 5 studies compared the effectiveness of local treatment (LT) in conjunction with ADT versus ADT alone, and the findings revealed that LT had more advantages in terms of OS and cancer-specific survival (CSS)^18^. Seisen et al.^19^ used the National Cancer Database (2003–2011) to identify 2967 individuals who received LT ± ADT versus ADT alone for cN1 PCa. Their results demonstrated that PCa patients with cN1 might benefit from any form of LT ± ADT over ADT alone. Furthermore, a meta-analysis has underscored a notably improved prognosis when abiraterone is combined with ADT, as compared to ADT in isolation, within the subset of patients afflicted by LNI and high-risk PCa^20^. This combination therapy should be deemed a novel standard of care.

Although numerous previous studies have discussed how to treat individuals with LNI after RP^21–23^, the equivalence of RP versus RT for initial treatment in patients with LNI remains uncertain. According to Sarkar et al.^24^*,* RP demonstrated no significant difference in CSM (HR: 0.47, 95% CI: 0.19–1.17, p = 0.1) or all-cause mortality (ACM) (HR: 0.88, 95% CI: 0.46–1.70, p = 0.71) compared to RT. Another study comparing RP ± ADT versus RT ± ADT showed no significant difference in OS between the two treatment modalities (HR: 0.54, 95% CI: 0.19–1.52, p = 0.2) after propensity score matching (PSM)^19^. In contrast, a study suggested that RP may confer a CSM advantage over RT. Specifically, after 1:1 PSM, 5-year overall mortality (OM) and CSM yielded respective multivariate HR of 0.63 (95% CI: 0.52–0.78, p < 0.001) and 0.66 (95%CI: 0.52–0.86, p < 0.001) for RP versus RT^25^. Given the lack of prospective research in LNI PCa, clinical patterns of practice vary widely. Currently, a prospective phase III randomized controlled study (RCT) (SPCG-15) is underway to compare RP ± RT with RT + ADT in locally advanced prostate cancer (LAPC)^26^. RT plays a significant role in prognostic models, and its importance in the initial treatment we have mentioned is noteworthy. Additionally, there was increasing evidence that combining adjuvant RT with ADT could increase survival in patients after RP when compared to ADT alone^27–29^. However, because these studies were conducted retrospectively, it was uncertain which patients would benefit the most. Our study may provide some reference for treatment modalities for PCa patients with LNI.

The use of non-statistical approaches or methods that do not involve statistical univariable pretesting of the relationships between candidate predictors and the result is a preferable strategy for selecting candidate predictors in multivariable modeling, according to the current bias assessment criteria (PROBAST: A Tool to Assess Risk of Bias and Applicability of Prediction Model Studies)^30^. During modeling, stepwise selection may be used to omit predictors. Therefore, in this study, the forward and backward stepwise selection was used to identify 8 prognostic factors including age, race, marital status, clinical T stage, GS, number of positive lymph nodes, RP, and RT. Although PSA level was not an independent prognostic variable (P = 0.281), we included it for the following reasons. Firstly, PSA level was included as a continuous variable, which may lead to statistical insignificance, but research had demonstrated that prognostication might be improved for clinical decision-making by utilizing continuous data rather than categorical data^31^. Secondly, PSA level was recognized as an independent prognostic variable^32^. Finally, machine learning algorithms take into account the possibility that other variables, which are not statistically significant, may yet have some influence on the prediction. This may be due to the algorithms' ability to forecast outcomes by examining inherent relationships between data that cannot be found using conventional statistical techniques. The results revealed that patients diagnosed at the T4 stage and with a GS ≥ 8 were independent prognostic factors. These findings were consistent with those of Zareba et al. and Abdollah et al.^33, 34^. We also found that the risk of death in patients increased with the number of positive lymph nodes. Compared with one positive lymph node, the risk was significantly higher in patients with more than two positive lymph nodes (HR: 1.631, 95% CI: 1.247–2.133, p < 0.001). The same conclusion was reached by Preisser et al.^35^. Furthermore, a new staging system based on the number of positive lymph nodes, derived by Daskivich's recursive partitioning analysis, also confirmed that as the number of positive lymph nodes increased, the patient's prognosis became worse^36^. Our study also discovered the effects of marital status on the prognosis of patients. Social support may have a substantial influence on cancer detection, treatment, and survival^37^. The prognostic impact of PCa patients was not significant in black people compared to white people, which was similar to a previous study^25^.

ML has advanced in tandem with computer technology, and its application has become ubiquitous across various industries. In addition, it has shown great potential for use in biomedical science^38^. The Cox regression, although widely used, has limited model flexibility. However, ML algorithms are not subject to non-proportionalities, multicollinearity, or nonlinearity^39^. Therefore, they can reduce the prediction bias caused by modeling uncertainty. It is noteworthy that while most ML analyses deal with classification problems and diagnostic models are developed using ML, it is more common in medicine to utilize survival analysis and develop prognostic models. Survival analysis is a kind of regression analysis. Its unique feature is that the training data is censored so that it can only be partially observed, which is different from ordinary regression analysis^40^. The goal of survival analysis, also known as time-to-event analysis or reliability analysis, is to establish a relationship between covariates and the time of an event. Some studies made the mistake of simply converting outcomes to categorical variables and using ML classification to develop prognostic models without considering the effect of censored data on the model, which biased the predicted risks. To avoid these pitfalls, we used scikit-survival for survival analysis and developed a prognostic model. Scikit-survival is a Python module for survival analysis that leverages the power of scikit-learn^16^.

When the goal is to predict the t-year risk of an event, the commonly utilized c-index for the time-to-event result is inappropriate. In the presence of a defined prediction interval, a misspecified model may have a higher c-index than a correctly specified model^41^. Scikit-survival also points out that if a specific time horizon is of primary interest (such as predicting death within n years), the c-index is not a useful performance measure. Therefore, in addition to using the c-index, we also used time-dependent AUC to evaluate model discrimination. Our findings indicated that no single algorithm outperformed the others consistently. While GBSA model had higher time-dependent AUC than the other models at most time points, EST model exhibited better performance at certain time points (Fig. 2). This illustrates that although the c-index is useful for evaluating overall performance, it may obscure intriguing features that only become apparent when examining time-dependent AUC at specific time points.

Several limitations to our study must be acknowledged. Firstly, our study's basis is a large retrospective cohort. Additional prospective clinical trials are still needed to obtain more precise results. Secondly, due to limitations within the SEER database, our analysis lacks information on the use of ADT. Touijer et al.^27^ investigated the impact of various postoperative management strategies on the outcomes of PCa patients with LNI, finding that there was no discernible difference in OS between patients who received ADT and those who did not, despite the ADT group exhibiting a reduced risk of CSM. Likewise, another study discovered no disparity in OS between those treated with ADT and those who were only monitored^42^, which may be related to the clinical condition, the pathological state, and the side effects of ADT. Our prognostic model fared admirably in internal validation, and the absence of ADT information had little bearing on our findings. While an analysis rooted in the SEER database offers marked progress over antecedent case-series reports, owing to its larger sample size, it does come at the expense of scant clinical particulars. Therefore, it becomes pivotal to amalgamate the broad-scale results presented herein with the finer-grained insights culled from prior analyses to holistically discern significant prognostic factors for prospective RCTs^43^. Thirdly, it merits emphasizing that the primary endpoint of our study singularly embraces late-stage survival, as epitomized by OS. Regrettably, owing to limitations inherent to the database, we were precluded from encompassing early-stage endpoints such as Progression-Free Survival (PFS). While OS unquestionably holds importance for PCa patients, especially those with prolonged life expectancies, it is plausible that including early-stage endpoints could provide a more comprehensive and nuanced prediction of prognosis from various angles. Fourthly, it is imperative to consider that the SEER database primarily draws from the US population. Hence, any extrapolation of our findings to other populations should be undertaken with caution. Lastly, our crucial aim for future research is to incorporate more independent external validation cohorts, a deficiency that we presently face.

## Conclusions

In summation, our study involved the development of prognostic models utilizing ML algorithms to predict OS in a cohort of 3280 PCa patients with LNI from the SEER database. Additionally, we created a web-based tool that can assist in identifying patients who may benefit from RP or RT and those who are at higher risk. This can aid physicians in making more informed decisions and providing individualized treatment for patients. Our research provides supporting evidence that ML algorithms hold immense potential for future clinical research and practice.

### Supplementary Information

Supplementary Information 1.

Supplementary Information 2.

## Data Availability

The data used in this study are available from the Surveillance, Epidemiology, and End Results Program (SEER) database (https://seer.cancer.gov/data/access.html).
