# Supplementary material for: Development of machine learning prognostic models for overall survival of prostate cancer patients with lymph node-positive
Source: Sci Rep. 2023 Oct 27;13:18424. doi: 10.1038/s41598-023-45804-x (PMC10611782; doi:10.1038/s41598-023-45804-x)
Supplement: Supplementary file 2 — Supplementary Information 2. [file 41598_2023_45804_MOESM2_ESM.docx]

**Supplementary Table 1.** Model parameter settings.

| **Models** | **Parameter** | **Value** |
| --- | --- | --- |
| Gradient Boosting Survival Analysis | n_estimators | 46 |
|  | learning_rate | 0.17 |
|  | min_samples_leaf | 3 |
|  | min_samples_split | 6 |
|  | max_depth | 3 |
|  | subsample | 0.27 |
|  | dropout_rate | 0 |
|  | random_state | 1 |
| Random Survival Forest | n_estimators | 36 |
|  | max_depth | 7 |
|  | min_samples_leaf | 11 |
|  | min_samples_split | 6 |
|  | random_state | 1 |
| Extra Survival Trees | n_estimators | 35 |
|  | max_depth | 8 |
|  | min_samples_leaf | 5 |
|  | min_samples_split | 2 |
|  | random_state | 1 |

The GBSA model and original data can be downloaded from the following publicly available GitHub repositories. ( <https://github.com/pengzihexjtu/PCa-N1>)


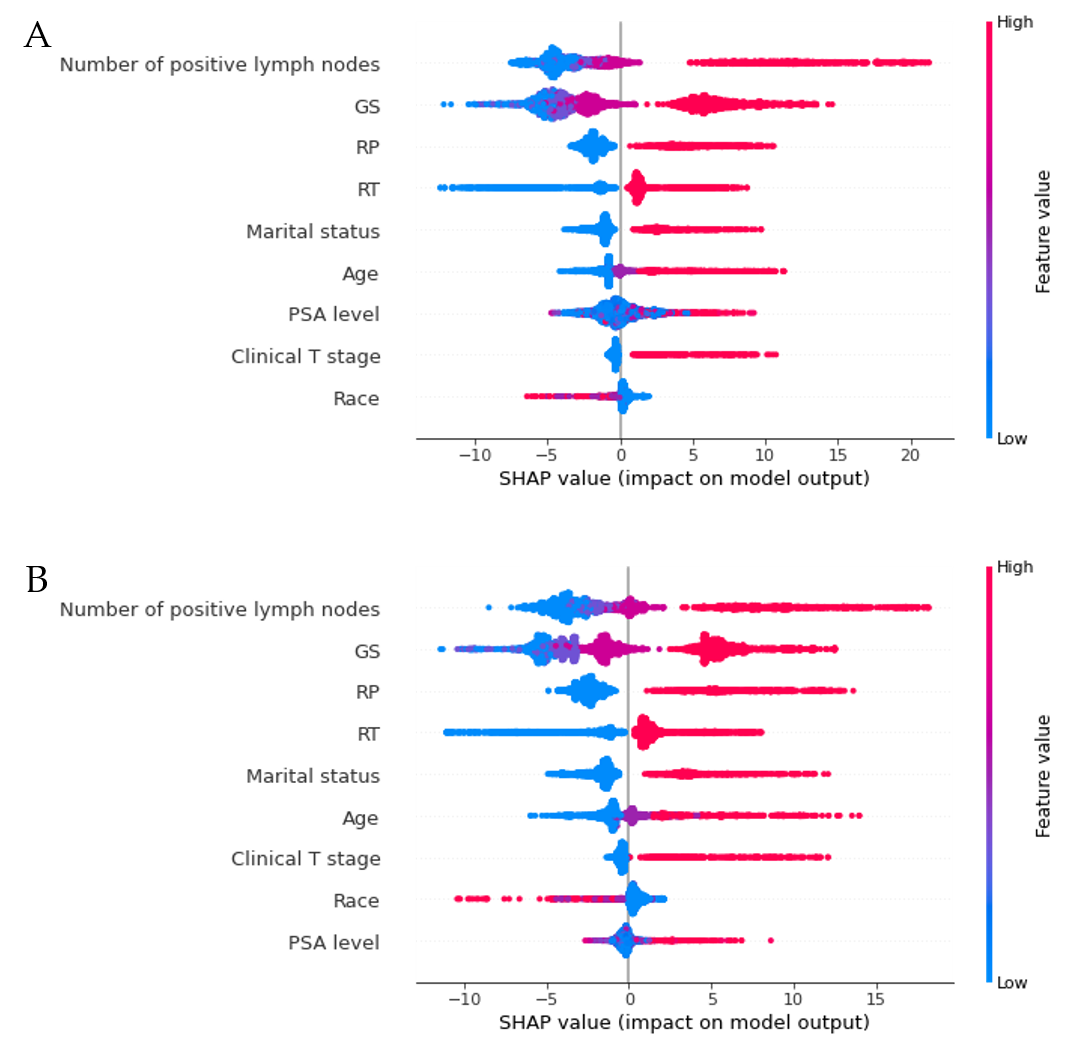


**Supplementary Figure 1.** The beeswarm plot. (A) The beeswarm plot of Random Survival Forest (RSF) model. (B) The beeswarm plot of Extra Survival Trees (EST) model.


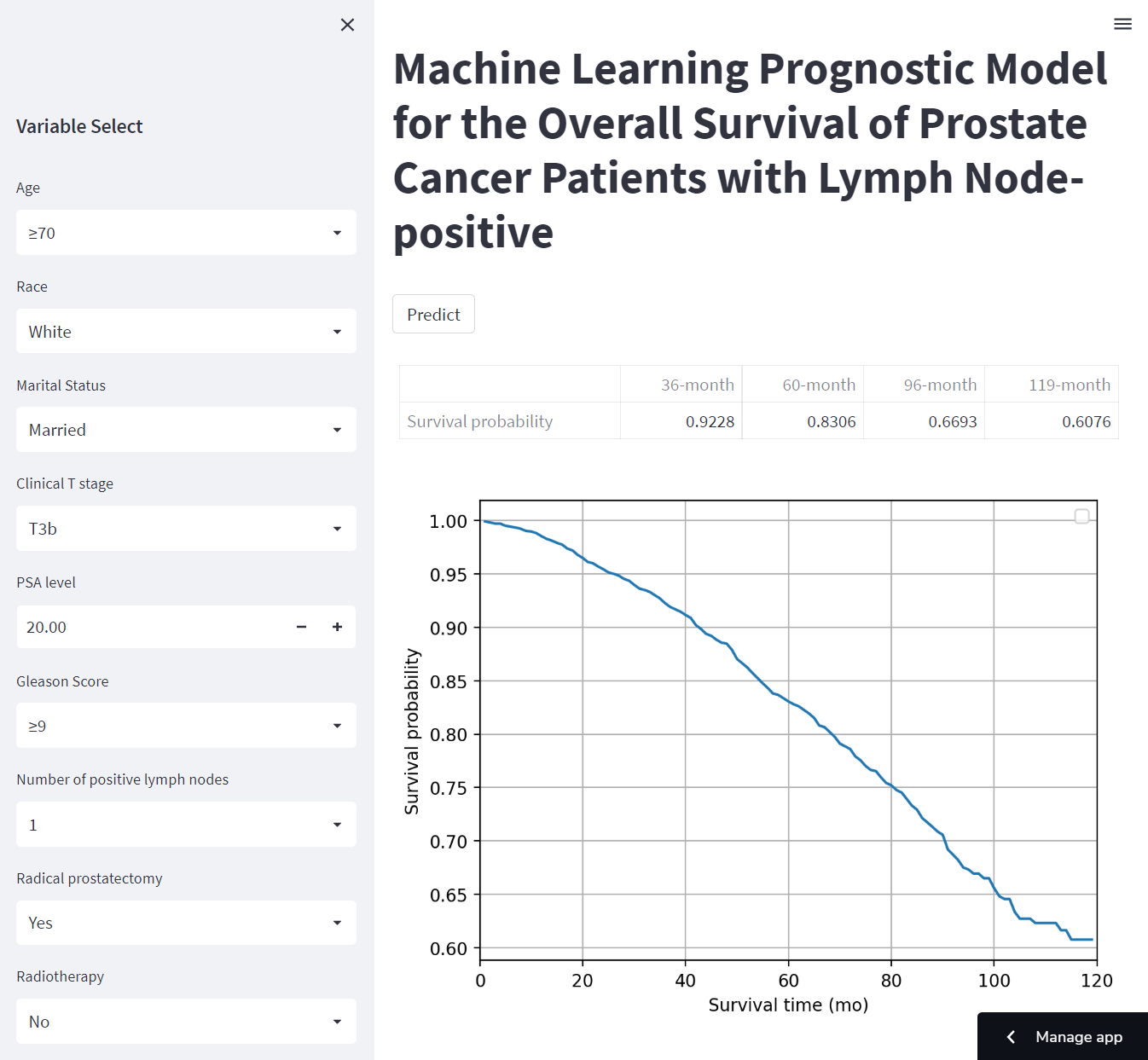


**Supplementary Figure 2.** The machine learning (ML) web-based predictor for predicting overall survival (OS) in prostate cancer (PCa) patients with lymph node-positive.
